# Teaching learning methods of an entrepreneurship curriculum

**Published:** 2015-10

**Authors:** KERAMAT ESMI, RAHMATALLAH MARZOUGHI, JAFAR TORKZADEH

**Affiliations:** Department of Educational Administration and Curriculum and Instruction, School of Education and Psychology, Shiraz University, Shiraz, Iran

**Keywords:** Teaching, Learning, Curriculum

## Abstract

**Introduction:**

One of the most significant elements of entrepreneurship curriculum design is teaching-learning methods, which plays a key role in studies and researches related to such a curriculum. It is the teaching method, and systematic, organized and logical ways of providing lessons that should be consistent with entrepreneurship goals and contents, and should also be developed according to the learners’ needs. Therefore, the current study aimed to introduce appropriate, modern, and effective methods of teaching entrepreneurship and their validation

**Methods:**

This is a mixed method research of a sequential exploratory kind conducted through two stages: a) developing teaching methods of entrepreneurship curriculum, and b) validating developed framework. Data were collected through “triangulation” (study of documents, investigating theoretical basics and the literature, and semi-structured interviews with key experts). Since the literature on this topic is very rich, and views of the key experts are vast, directed and summative content analysis was used. In the second stage, qualitative credibility of research findings was obtained using qualitative validation criteria (credibility, confirmability, and transferability), and applying various techniques. Moreover, in order to make sure that the qualitative part is reliable, reliability test was used. Moreover, quantitative validation of the developed framework was conducted utilizing exploratory and confirmatory factor analysis methods and Cronbach’s alpha. The data were gathered through distributing a three-aspect questionnaire (direct presentation teaching methods, interactive, and practical-operational aspects) with 29 items among 90 curriculum scholars. Target population was selected by means of purposive sampling and representative sample.

**Results:**

Results obtained from exploratory factor analysis showed that a three factor structure is an appropriate method for describing elements of teaching-learning methods of entrepreneurship curriculum. Moreover, the value for Kaiser Meyer Olkin measure of sampling adequacy equaled 0.72 and the value for Bartlett’s test of variances homogeneity was significant at the 0.0001 level. Except for internship element, the rest had a factor load of higher than 0.3. Also, the results of confirmatory factor analysis showed the model appropriateness, and the criteria for qualitative accreditation were acceptable.

**Conclusion:**

Developed model can help instructors in selecting an appropriate method of entrepreneurship teaching, and it can also make sure that the teaching is on the right path. Moreover, the model is comprehensive and includes all the effective teaching methods in entrepreneurship education. It is also based on qualities, conditions, and requirements of Higher Education Institutions in Iranian cultural environment.

## Introduction

Nowadays, entrepreneurship has turned into one of the most significant university activities. Moreover, its key role in progress of societies has made many universities in developed and developing countries take utilizing entrepreneurship into account. Also, by developing strategies, policies, and scientific programs, they can step towards lifting and reinforcing students’ entrepreneurship spirit and behavior through effective training (1). Training in entrepreneurship is an activity utilized to communicate knowledge and information required for setting and running businesses. In addition, it enhances, improves, and develops non-entrepreneurs’ attitudes, skills, and abilities(2). Training in entrepreneurship affects the level of trends, activities, and entrepreneurial passion, as a result of which setting and developing new businesses in economy is also affected (3).

According to Kitz (1991), identifying the most effective methods for introducing teachable skills and relationships between the learners’ needs and teaching methods are the keys to a successful entrepreneurship education. In this regard, Jack and Anderson (1998) considered entrepreneurship education as a combination of science and art. That is, its science part relates to functional skills required for starting a business, and the art-related part is concerned with creative aspects of entrepreneurship. Moreover, even entrepreneurship instructors unanimously agree upon the necessity for paying simultaneous attention to scientific and artistic aspects of entrepreneurship and its teaching using creative methods (4).

With regard to entrepreneurship teaching methods, no certain method is offered. An overview of the literature on entrepreneurship shows the change pattern from conventional teaching of entrepreneurship to modern methods based on “action learning”.  Entrepreneurship education is, simply defined, a systematic, conscious, and goal-oriented process, through which non-entrepreneur individuals who has the necessary potential are creatively trained. In fact, entrepreneurship education is an activity used to transfer knowledge and information required for entrepreneurship and leads to increase in, improvement, and development of non-entrepreneurs’ attitudes, skills, and abilities. Moreover, it forms the students’ beliefs and values for creating an entrepreneurship culture (5).

Entrepreneurship education should be considered as a creative process rather than a mechanical one. Therefore, teaching such a process would be challenging and problematic and requires new and active teaching methods. Researchers have offered various methods for teaching entrepreneurship. Oyelola (2013) offers process-oriented teaching instead of content-oriented, problem-based teaching instead of introducing concepts and methods such as group project, writing business plans, practical experience in producing and selling products and services, and learning from mistakes (6). Arasti et al. (2012) think of group project, case study, individual projects, developing a new investment project, problem solving, guiding young entrepreneurs by supporting them in their projects, training in investment, group discussion, official speech, interviewing entrepreneurs, simulations, and scientific visits as the most important methods of teaching entrepreneurship (7).

Torben (2010) thinks of entrepreneurship camps as teaching methods (8). Potter (2008) emphasizes teaching methods of business planning, case studies, students’ starting businesses, business games, student entrepreneurs’ teams and networks, internship in small companies, feasibility studies, training in communication, getting advice in starting small businesses, distance education, and external cooperation and offers business simulations, games, analysis and discussion of real businesses, group work, mentoring, networking (common experience), tutoring, action learning, problem-oriented method, peer group support, expert advice and intensive counseling, and access to business networks as teaching methods of entrepreneurship (9, 10).

Solomon (2008) also emphasizes the case study, business planning, discussions, research projects, computer simulations, entrepreneurship, visiting sites and class practice (11). With regard to entrepreneurship education, Ton and Frank (2006) focus on problem-oriented learning, active learning, and action learning (12). Sharif et al. (2011) introduced entrepreneurship through individual, extracurricular activities and entrepreneurship training, defining and completing individual projects, action research, probing process, turning ideas into action plans, discussing important and perfect actions plans and implementing designed plans, control over research methods, and scientific, educational, and research development (13).

According to Mojalal et al. (2011), utilizing problem solving, active training methods, and practical learning activities, presenting creativity opportunities, developing new ideas, and holding classes and specialized workshops can be offered as entrepreneurship teaching methods (14). Also, Kowsari and Norouzzadeh (2009) considered experiences in real life and assimilated learning environments, group work, involving in learning, action research, and permanent and continuous connection with entrepreneurs as teaching methods (15). In addition, according to Yadollahi et al. (2009) emphasized on practical teaching methods, workshops, seminars, interview, visiting entrepreneurs and speeches (16).

Taking into account all the previous studies and methods introduced by different researchers, analyzing methods of entrepreneurship teaching and recognizing their strengths and weaknesses methods, we aimed to offer a clear and comprehensive classification of the methods that best suit educational and cultural structure of Iranian universities. Therefore, to introduce these methods, present educational and technological facilities, university culture and communication, characteristics of teachers and learners, and budgets should be taken into consideration. 

## Methods

The goal of the current study is of a fundamental- developmental kind and the results have practical recommendations. Since the current research seeks to establish new thoughts and researches in the field of teaching-learning methods of entrepreneurship, and also aims to explain unknown aspects of the model, it is considered as fundamental-developmental. Also, it is practical because it introduces practical and appropriate methods to instructors. With regard to research method, we used mixed method. It is also of a sequential exploratory type, through which qualitative data and then quantitative data were gathered. Finally, both qualitative and quantitative analyses were interpreted simultaneously (17). The present study was conducted in two phases:

- Development of teaching-learning methods of entrepreneurship curriculum and its elements: In this phase of the study, teaching-learning methods of entrepreneurship curriculum and its elements are developed on the basis of Content Analysis, triangulation, and three data resources, namely theoretical bases, research and practical records, key experts (curriculum planners, entrepreneurs, entrepreneurship instructors, higher education planners, etc.)
- Validation of the developed framework: In this phase of the study, field data were gathered from available sample. Then, teaching-learning methods of entrepreneurship curriculum are validated through exploratory and confirmatory Factor Analysis.

Subsequently, the two phases will be addressed in more details.

**Development of entrepreneurship curriculum teaching-learning methods and their elements**

By studying specialized sources and references about entrepreneurship education, theoretical basics, previous studies, and exploiting views of the experts in this stage, teaching-learning methods of entrepreneurship curriculum and their elements were determined via triangulation procedure. Since the researcher was faced with vast and various views of the experts, and also with rich, sophisticated, highly distributed, and multi-dimensioned data, data coding and classification, using qualitative factor analysis, was necessary. Therefore, open, axial, and selective coding were applied on data. In this regard, firstly initial data (researcher’s suggested principles and interviewees’ expert opinions) were precisely investigated and open codes were exploited. Then, after repeated and continuous corrections, classifications were formed and compared with each other; in the case of need, they were either combined or in some cases one classification was divided into two or more classifications, or in some cases the place of a code was changed in order for core classifications (teaching-learning methods) to be obtained. During analyses, it was found that most of the experts considered three teaching-learning methods (direct, interactive, and practical-operational) as the main teaching methods. Also, according to their unanimous opinions, elements of these methods (30 elements) were recognized after necessary corrections were made (Table 1).

**Table 1:** Teaching-learning methods of entrepreneurship curriculum

**Teaching-learning methods**	**Elements**
**Direct teaching-learning methods**	Inviting guest entrepreneurs – Mentoring - Official speech-seminars – Video watching and recording - Training in extracurricular activities -Training in specialized lessons - Small businesses mentoring –Entrepreneurship tutoring
**Interactive teaching-learning methods**	Process-oriented learning - Learning from mistakes - Interviewing entrepreneurs - Bilateral learning - Group discussion - Networking – Discussion - Problem-oriented learning - Actice learning
**Practical-operational teaching-learning methods**	Role-playing - Training workshops - Site visiting - Class practice -Research projects – Internship - Business planning - Starting business - Studying nature - Investment projects - Practical experience

**Validation of developed framework**

- **Qualitative reliability and validity**
In order to evaluate the data quality credit, qualitative validation criteria including “credibility, confirm ability, transferability” were used (emphasized by researchers such as Andres, 2003; Rao and Perry, 2003; Guba and Linkoln, 1994) (18). In order to determine data credibility techniques of triangulation, obtaining precise and parallel data, member controls, and researcher self-reviewing, transferability techniques of achieving theoretical saturation, using specialized coding and symbol analysis procedures, and comprehensive data description, and finally the  confirmability, these techniques were utilized: data collection from diverse resources, flexibility of proposed theoretic framework, analysis of negative cases, and method flexibility. Also, in order to make sure that the research qualitative part is reliable, reliability tests were utilized. It was conducted through testing and documenting investigation processes by means of documents and sufficient evidence.
- **Quantitative reliability and validity**
  In order to evaluate the framework quantitative reliability and validity, the use was made of factor analysis and Cronbach’s Alpha coefficient. It was conducted by turning the teaching-learning methods and their elements into a questionnaire and then giving it to 90 key experts selected through purposive sampling. Afterwards, by utilizing data, the framework factor structure was investigated by means of exploratory factor analysis, and then the pattern resulting from exploratory factor analysis was investigated and its fitting was calculated through confirmatory factor analysis. The reliability was calculated using Cronbach’s Alpha, whose value was 0.83.

## Results

The results of exploratory factor analysis, which was conducted by means of main elements and Varimax rotation, showed that a three factor structure would be an appropriate method of describing elements concerning the teaching-learning entrepreneurship curriculum. The pattern obtained from exploratory factor analysis was examined using LISREL software, confirmatory factor analysis and also fit indexes such as Chi-square, goodness of fit, goodness of comparative fit, comparative fit, Normed-fit,  Non-Normed-fit, and root mean square deviation, as a result of which the best fit was obtained.

These three factors wholly explain 54.79 percent of the scale variance. It should be noted that the calculated value is acceptable. On the basis of content items, the three factors are direct, interactive, and practical-operational methods of presentation, respectively. The mentioned factors encompass some elements (teaching methods), all of which have higher than 0.3 per cent factor loading on their factors. Teaching methods of direct presentation include inviting guest entrepreneurs, tutoring entrepreneurship, presenting official speech, holding seminars, watching and recording videos, counseling, and training through extracurricular activities.

Interactive teaching methods are active learning, discussion, networking, group project, group discussion, bilateral learning, learning from mistakes, and process-oriented teaching.

Practical-operational teaching methods include practical experience, study of nature, starting businesses, business plans, research projects, class practice, workshops, and role play.

It should be noted that due to factor loading value of less than 0.3 per cent, internship element, which belongs to practical-operational group, was deleted from the scale.

It should be noted that the value of KMO test of sampling adequacy equaled 0.72, and the value of Bartlett's Test of Sphericity measuring significance of correlation matrix was significant at the level of 0.001. Questionnaire reliability with a significance level of 0.99 and value of 0.83 was calculated using Cronbach’s Alpha.

In order to confirm the model resulting from exploratory factor analysis, and to determine its fitting, we used confirmatory factor analysis. It was conducted utilizing LISREL software. According to the results, the value of obtained factor loadings was higher than 0.3, as the result of which the identified elements were good indicators of the aspects (direct, interactive, and operational-practical methods). The model fitting indexes indicated its appropriate and perfect fitting (Table 2).

**Table 2:** Fitting indexes related to confirmatory factor analysis of teaching-learning methods of entrepreneurship curriculum

**Pattern**	X2	df	X2/df	RMSEA	GFI	AGFI	NFI	CFI
**Main pattern (without correction)**	665.64	375	1.77	0.093	0.66	0.61	0.67	0.77
**Main pattern (with correction)**	484.58	344	1.40	0.068	0.82	0.77	0.91	0.87
**Optimal value**			<2	<0.08	>0.9	>0.9	>0.9	>0.9

As shown in Table 2, by making some corrections (freeing the path of Training through Extracurricular Activities from group discussion, networking from learning by mistakes, and site visiting from role playing), fitting indexes reached an acceptable level. It shows that the pattern obtained from exploratory factor analysis (especially, after making corrections) has an optimal fitting (Table 3).

**Table3:** Confirmatory factor analysis table of corrected pattern for the scale of teaching-learning methods of entrepreneurship curriculum

**Teaching-learning methods**	**Elements**	**Factor loading**
Direct teaching-learning methods	Inviting guest entrepreneurs Mentoring Official speech Seminars Video watching and recording Training in extracurricular activities Training in specialized lessons Small businesses mentoring Entrepreneurship tutoring	0.61 0.59 0.74 0.31 0.50 0.74 0.76 0.87 0.56
Interactive teaching-learning methods	Process-oriented learning Learning from mistakes Bilateral learning Group discussion Networking Discussion Problem-oriented learning Discussion	0.87 0.42 0.76 0.83 0.44 0.89 0.68 0.89
Practical-operational teaching-learning methods	Role-playing Training workshops Site visiting Class practice Research projects Internship Business planning Starting business Studying nature Investment projects Practical experience	0.79 0.84 0.62 0.67 0.69 0.39 0.96 0.68 0.69 0.46 0,76

According to the findings shown in the above Table, due to having a factor loading higher than 0.3, the items of the three teaching methods (direct presentation, interactive, and practical-operational) were favorable measurements of theses dimensions. Also, the obtained embedding index shows that the model enjoys a favorable embedding. 

## Discussion

By meta-analyzing the existing entrepreneurship teaching methods and identifying their weak and strong points, the current study aimed to introduce a clear and comprehensive classification of the methods appropriate to educational and cultural structure of Iranian universities, existing technologies and educational facilities, university culture and communications, budget and technology facilities and professors’ and learners’ characteristics. In this regard, conducting various studies, many researcher have sought to refer to the existing deficiencies and defects, and at the same time they have offered new methods. But the point is that all of these studies have focused on curriculum multiple elements; that is, none of them have taken teaching methods into account as a special and significant topic. 

Identifying conditions of entrepreneurship education in the humanities, Safari et al. (2012) showed that in almost all the humanities disciplines, material, content, and teaching methods are not in a favorable condition for communicating entrepreneurship and business concept (19). Moreover, Yadollahi et al. have prioritized main materials, skills, educational goals, and the best learning-teaching methods of entrepreneurship education in educational sciences (16). Moreover, Graevenitz et al. (2010), Shamsollahi (2012), Ahmadzadeh (2005), and Molamohammadi (2000) have studied entrepreneurship curriculum, none of which has been able to present a comprehensive classification of the curriculum with regard to the nature and approach of employing methods such as what the present study has conducted (20-23).

## Conclusion

The pattern obtained from this research indicates teaching-learning methods of entrepreneurship curriculum which can help the instructors select an appropriate method of teaching entrepreneurship in class and make sure that the teaching process is on the right path. Since the pattern uses reliable data and quantitative and qualitative validation methods, it is credible and its findings can be relied. Moreover, due to comprehensiveness, the pattern includes all the effective teaching methods of entrepreneurship education system. It is also proportional to Iranian culture leading to diversity of educational opportunities in academic centers for meeting the employment needs of learners. Using this model, instructors can select an appropriate teaching method, and design their educational activities in accordance with it. Also, by utilizing these teaching methods, learners can participate in more challenging educational activities, and consequently gain experiences which provide some insights into discovering and creating entrepreneurship opportunities. Moreover, it enables them to start and manage their businesses successfully and also take advantage of these educational opportunities. 
